# In Situ Preparation and Analysis of Bimetal Co-doped Mesoporous Graphitic Carbon Nitride with Enhanced Photocatalytic Activity

**DOI:** 10.1007/s40820-018-0236-y

**Published:** 2019-01-23

**Authors:** Wanbao Wu, Zhaohui Ruan, Junzhuo Li, Yudong Li, Yanqiu Jiang, Xianzhu Xu, Defeng Li, Yuan Yuan, Kaifeng Lin

**Affiliations:** 1 School of Chemistry and Chemical Engineering, Harbin Institute of Technology, 92 West Dazhi Street, Nan Gang District, Harbin, 150001 People’s Republic of China; 2 Key Laboratory of Aerospace Thermophysics, Ministry of Industry and Information Technology, Harbin Institute of Technology, 92 West Dazhi Street, Nan Gang District, Harbin, 150001 People’s Republic of China

**Keywords:** Co-doped g-C_3_N_4_, Porous g-C_3_N_4_, Photocatalysis, Optical simulation, Light absorption intensity

## Abstract

A novel photocatalyst of mesoporous graphitic carbon nitride (g-C_3_N_4_) co-doped with Co and Mo (Co/Mo-MCN) has been one-pot synthesized via a simple template-free method; cobalt chloride and molybdenum disulfide were used as the Co and Mo sources, respectively. The characterization results evidently indicate that molybdenum disulfide functions as Mo sources to incorporate Mo atoms in the framework of g-C_3_N_4_ and as a catalyst for promoting the decomposition of g-C_3_N_4_, resulting in the creation of mesopores. The obtained Co/Mo-MCN exhibited a significant enhancement of the photocatalytic activity in H_2_ evolution (8.6 times) and Rhodamine B degradation (10.1 times) under visible light irradiation compared to pristine g-C_3_N_4_. Furthermore, density functional theory calculations were applied to further understand the photocatalytic enhancement mechanism of the optical absorption properties at the atomic level after Co- or Mo-doping. Finite-difference time-domain simulations were performed to evaluate the effect of the mesopore structures on the light absorption capability. The results revealed that both the bimetal doping and the mesoporous architectures resulted in an enhanced optical absorption; this phenomenon was considered to have played a critical role in the improvement in the photocatalytic performance of Co/Mo-MCN.

**Electronic supplementary material:**

The online version of this article (10.1007/s40820-018-0236-y) contains supplementary material, which is available to authorized users.

## Highlights

- Co/Mo co-doped mesoporous graphitic carbon nitride (g-C_3_N_4_) exhibited enhanced photocatalytic performances with regard to H_2_ generation (8.6 times) and Rhodamine B degradation (10.1 times) compared with pristine g-C_3_N_4_.
- The density functional theory calculations and optical simulation illustrate that Co/Mo co-doping and the created mesoporous structure can enhance light absorption.
- The enhanced activity depends on the synergistic effect of Co and Mo co-doping.

**Electronic supplementary material:**

The online version of this article (10.1007/s40820-018-0236-y) contains supplementary material, which is available to authorized users.

## Graphical abstract

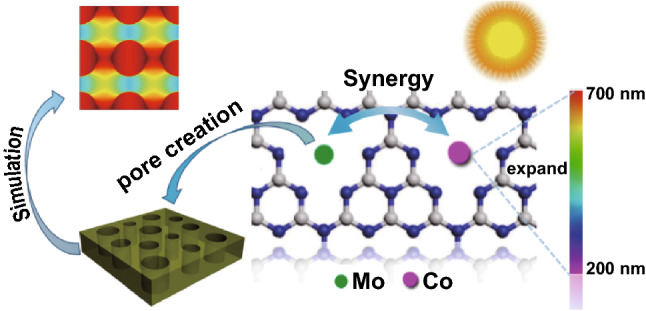

## Introduction

The energy crisis and environmental pollution are two significant global challenges [1–3]. Water-splitting to produce clean hydrogen through solar energy conversion is considered to be a sustainable and feasible solution to address these challenges [4–6]. In recent years, the emergent semiconductor photocatalytic technology has attracted substantial attention for realizing organic pollutant degradation and solar energy conversion [7–10]. As a new non-metallic semiconductor, graphitic carbon nitride (g-C_3_N_4_) has attracted substantial attention; it exhibits advantages such as high thermal stability, non-toxicity, abundant precursors, and a suitable bandgap that could expand the absorbing threshold up to 460 nm [11–13]. However, drawbacks of pristine g-C_3_N_4_ (P-CN) such as the low specific surface area and rapid recombination rate of photoexcited charge carriers results in its low photocatalytic efficiency [14–16]. Various strategies have been adopted to modify photocatalyst in order to improve photocatalytic performance, such as metal doping, pore creating, and semiconductor coupling [17–20]. Combining two or three of these strategies would improve photocatalytic activity more remarkably [21–23].

A mesoporous structure and high specific surface area also play vital roles in photocatalysis; they can result in improved photocatalytic efficiency owing to the promoted separation of photogenerated active charges and improved mass transfer. Generally, hard and soft template methods are applied to synthesize porous g-C_3_N_4_ with various pore structures [13, 24, 25]. However, certain drawbacks of the template methods limit their wide application. The synthesis process of the hard template method is complex and tedious, resulting in increased costs; moreover, the removal of a few silica templates by using the aqueous ammonium bifluoride (NH_4_HF_2_) or hydrogen fluoride (HF) negatively impacts the environment. The porous g-C_3_N_4_ prepared via soft template method would result in a higher carbon residue causing the “shielding effect”; this reduces light absorption and results in low catalytic activity [26–28]. Compared to hard or soft template, the template-free method does not require other substances as templates; furthermore, mesopores can be made by selecting suitable precursors [29–31]. Therefore, the template-free method as a convenient strategy to develop mesopores is highly desirable.

In addition, the g-C_3_N_4_ can function as electron donors to trap transition metal ions to form metal-N bonds owing to its heptazine ring with pyridinic nitrogen and six nitrogen lone pairs of framework [32]. Several previous studies on transition metal-doped g-C_3_N_4_ demonstrated its superior photocatalytic performances [33–35]. Wang et al. [36] studied Fe-doped g-C_3_N_4_ with enhanced photocatalytic activity for Rhodamine B (RhB) degradation. The study by Oh et al. [32] demonstrated that metal-doped g-C_3_N_4_ (Cu, Co, and Fe) catalysts can improve the catalytic activity of sulfathiazole degradation; moreover, the Co-doped g-C_3_N_4_ presented the highest catalytic activity. However, few efforts have focused on the bimetallic doping of g-C_3_N_4_, which is likely to exhibit more efficiency in photocatalytic reaction owing to the possible synergistic effect of the doped bimetal [37]. Inspired by the work that reported the synthesis of Cr^3+^ and Ce^3+^ co-doped N/TiO_2_ and its superior photocatalytic performance for the degradation of humic acid owing to the red shift of the bandgap energy [38], it could be speculated that bimetallic doping of g-C_3_N_4_ is of significant interest.

Herein, co-doped mesoporous g-C_3_N_4_ with Co and Mo elements (Co/Mo-MCN) was prepared via a simple one-pot method by using cobalt chloride (CoCl_2_) as the Co source and MoS_2_ as the Mo source; its photocatalytic activity was evaluated by hydrogen evolution and RhB degradation under visible light irradiation. To our knowledge, this is the first study to prepare g-C_3_N_4_ with bimetal co-doping and to construct mesopores via a template-free approach. Compared with pristine g-C_3_N_4_ (P-CN) and mono-metal-doped g-C_3_N_4_, the as-prepared Co/Mo-MCN presented significantly higher photocatalytic activity. Furthermore, first principle calculations and optical simulations are carried out to analyze the electronic structure and optical absorption characteristics of the P-CN and doped g-C_3_N_4_. This study proposes a new method for the design and synthesis of a g-C_3_N_4_ with co-doping metals and structured mesopores; it also investigates the effect of the co-doping and the mesopores on the light absorption capacity, through finite-difference time-domain (FDTD) simulation and density functional theory (DFT) calculation. Furthermore, a feasible mechanism for enhanced photocatalytic of bimetal co-doped g-C_3_N_4_ is recommended.

## Experimental

### Materials

#### Synthesis of Pristine g-C_3_N_4_

The P-CN was prepared by directly calcinating 8.0 g of guanidine hydrochloride at 550 °C for 60 min in a muffle furnace at a heating rate of 3 °C min^−1^.

#### Synthesis of Co-doped g-C_3_N_4_

Co-doped g-C_3_N_4_ (Co-CN) was synthesized by calcinating guanidine hydrochloride and Co precursor according to the modified method in the literature [39]. Briefly, 8.0 g of guanidine hydrochloride and a certain amount of CoCl_2_·6H_2_O (5, 10, 30, and 100 mg) were dissolved in 5 mL of deionized water. After stirring for 1 h, the mixture was dried at 80 °C for 1 day. The solids obtained were then grinded into powder and transferred to a quartz crucible; the sintering process was similar to that for P-CN. The products were denoted as Co-CN-5, Co-CN-10, Co-CN-30, and Co-CN-100, respectively.

#### Synthesis of Mo-Doped Mesoporous g-C_3_N_4_

Mo-doped mesoporous g-C_3_N_4_ (Mo-MCN) was prepared by using MoS_2_ nanosheets as the Mo precursor, which were obtained via hydrothermal method. In a typical run, 1.694 g of sodium molybdate and 2.665 g of thiourea were dissolved in 24 mL of deionized water and then transferred to a stainless autoclave for 24 h at 220 °C. After being cooled naturally, the obtained black product was washed with ethanol and deionized water for several times until the supernatant was clear; then, it was dried at 60 °C. The final product was formulated into a 1 mg mL^−1^ MoS_2_ ethanol suspension and sonicated for 6 h. The typical synthesis of Mo-MCN samples was as follows: 8.0 g of guanidine hydrochloride and MoS_2_ solution (1, 2, and 4 mL; 1 mg mL^−1^) were mixed with 5 mL of deionized water. After stirring for 0.5 h and sonication treatment for 1 h, the mixture was dried at 80 °C for 24 h. Then, it was grinded into powder and heated at 550 °C for 1 h in air. The products were labeled as Mo-MCN-1, Mo-MCN-2, and Mo-MCN-4, respectively.

#### Synthesis of Co and Mo Co-doped Mesoporous g-C_3_N_4_

Co/Mo-MCN was prepared through the following typical experiment: 2 mL of MoS_2_ ethanol solution (1 mg mL^−1^), 8.0 g of guanidine hydrochloride, and a desired amount of CoCl_2_·6H_2_O (3, 5, 10, and 50 mg) were added to 5 mL of deionized water. After stirring for 0.5 h and sonication treatment for 1 h, the mixture was dried at 80 °C for 24 h. Then, it was grinded into powder and heated at 550 °C for 1 h in air. The products were denoted as Co/Mo-MCN-3, Co/Mo-MCN-5, Co/Mo-MCN-10, and Co/Mo-MCN-50, respectively.

### Photocatalytic Activity Measurement

The photocatalytic activity of the samples was examined by RhB degradation and hydrogen generation under visible light irradiation (> 400 nm). The H_2_ production was performed in a closed system. Typically, a 100 mg of photocatalyst was dispersed in 300 mL of aqueous solution containing TEOA (10 vol%), and Pt (1.5 wt%). After 5 min of sonication, the solution was degassed under flowing N_2_. A 300-W Xe lamp equipped with UV filter (> 400 nm) was used as a light source. Evolved gas of 1 mL was sampled for analysis by gas chromatography with a TCD detector. For degrading the RhB solution, 50 mg of photocatalyst was dispersed in 50 mL of RhB solution under stirring. Considering the adsorption of the photocatalysts, the suspension was kept in dark for 30 min to attain an adequate adsorption–desorption equilibrium prior to irradiation.

## Results and Discussion

**Fig. 1:**
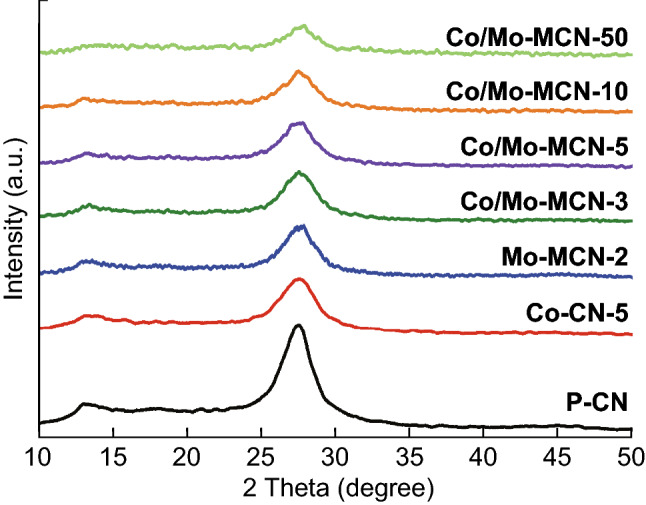
XRD patterns of P-CN, Co-CN-5, Mo-MCN-2, and Co/Mo-MCN composites

The XRD patterns of the prepared P-CN, Co-CN, Mo-MCN, and Co/Mo-MCN are shown in Figs. 1 and S1. All the samples display two characteristic peaks at 13.1° and 27.5°, belonging to the (100) plane and (002) plane of g-C_3_N_4_, respectively [40, 41]. No other peaks are observed in any of the as-prepared samples, indicating the absence of Co compound and Mo compound [19, 42]. In addition, the peak intensities of the (002) plane in the Co-CN and Co/Mo-MCN weakened and broadened with the increase in the Co contents; moreover, those in the Mo-MCN also gradually weakened with the increase in the Mo contents. This phenomenon can be attributed to the introduction of Co and/or Mo, which inhibits the thermal polymerization of g-C_3_N_4_ [39, 43]. The XRD pattern of MoS_2_ nanosheets is presented in Fig. S2; the diffraction peaks at 14.10°, 33.23°, 39.45°, 49.01°, and 58.66° corresponding to the (002), (100), (103), (105), and (110) planes of the hexagonal phase of MoS_2_ (JCPDS 37-1492) [44, 45]. Figure S3 display SEM images of MoS_2_. They are nanoflowers (Fig. S3a, b) prior to ultrasound and nanosheets (Fig. S3c, d) after ultrasound. Figure S4 displays the FT-IR spectra of P-CN, Co-CN, Mo-MCN, and Co/Mo-MCN. All the characteristic peaks of these samples are similar to that of P-CN, indicating that the framework structure of P-CN was not changed after the doping with Co or/and Mo [46, 47]. Notably, the characteristic peaks of Co–N or Mo–N bonds are not observed; this implied that Co or Mo atoms were absent in the materials or that their mass ratios are negligible to be distinguished from the characteristic peaks of g-C_3_N_4_.

**Fig. 2:**
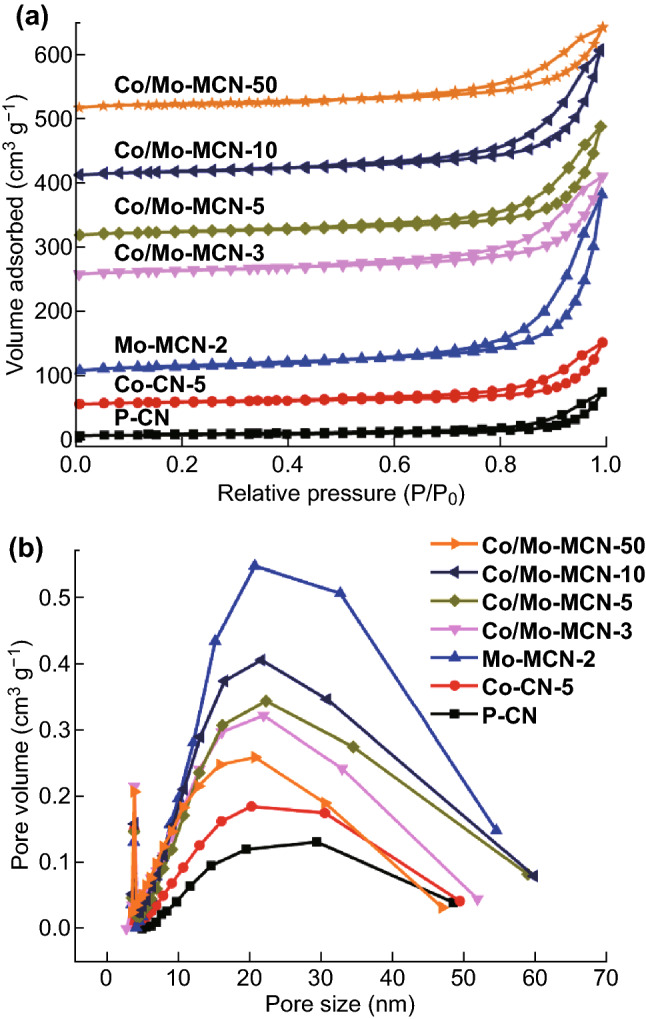
**a** Nitrogen adsorption–desorption isotherms and **b** corresponding pore size distribution curves of P-CN, Co-CN-5, Mo-MCN-2, and Co/Mo-MCN

The nitrogen adsorption–desorption was carried out to monitor the specific surface areas and porous structures of the as-prepared materials (Figs. 2 and S5). The isotherms of all the samples display hysteresis loops (type IV with H_3_ hysteresis loops) at a higher relative pressure range (0.8–1.0), implying the presence of mesopores [48–50]. P-CN exhibits low *S*_BET_ and pore volume **(**Table S1). For Co-CN, the *S*_BET_ and pore volume are unchanged after the Co-doping. However, the *S*_BET_ and pore volume of Mo-MCN significantly increase after the amount of Mo-doping increases; moreover, Mo-MCN-2 exhibits the highest *S*_BET_ (82.4 m^2^ g^−1^) and pore volume (0.44 cm^3^ g^−1^). Notably, the product is completely decomposed when the content of MoS_2_ exceeds 4 mg. The enlarged *S*_BET_ and pore volume can be ascribed to the inhibition of the crystal growth by Mo-doping and substantial gas evolution (such as NH_3_, CO_2_, and H_2_S) owing to the decomposition of g-C_3_N_4_ caused by the presence of MoS_2_ to create more mesopores [51]. Furthermore, all the Co/Mo-MCN exhibit significantly higher *S*_BET_ (approximately 60 m^2^ g^−1^) than that of P-CN (16 m^2^ g^−1^). Figure 2b shows that the pore size distribution of all the materials are centered at 20–30 nm. A similar mesoporous structure and high specific surface area are favorable for the mass transfer of the reactants and for increasing the efficiency of charge carrier separation, thereby improving the catalytic activity.

**Fig. 3:**
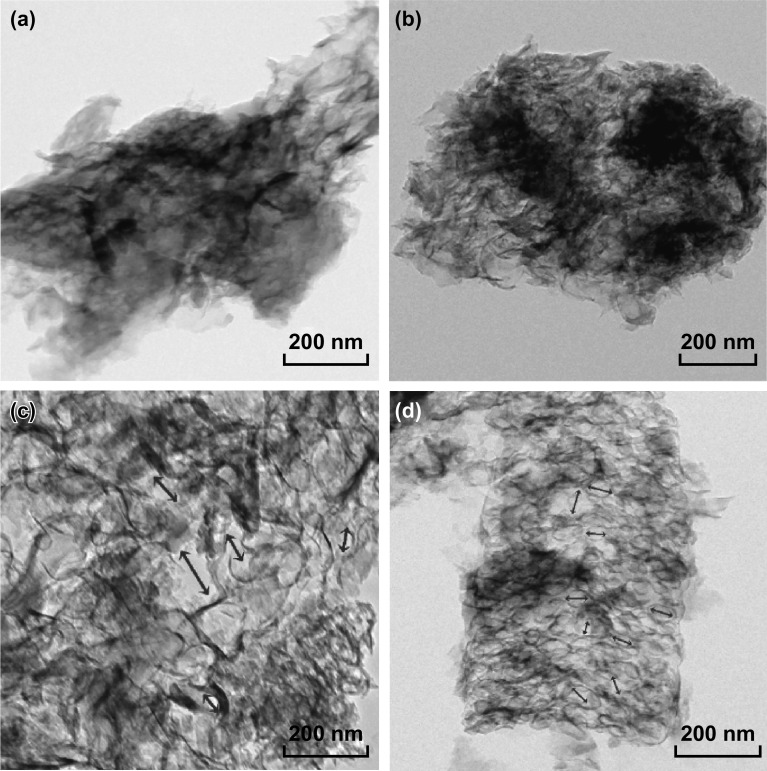
TEM images of **a, b** P-CN and **c, d** Co/Mo-MCN-5

**Fig. 4:**
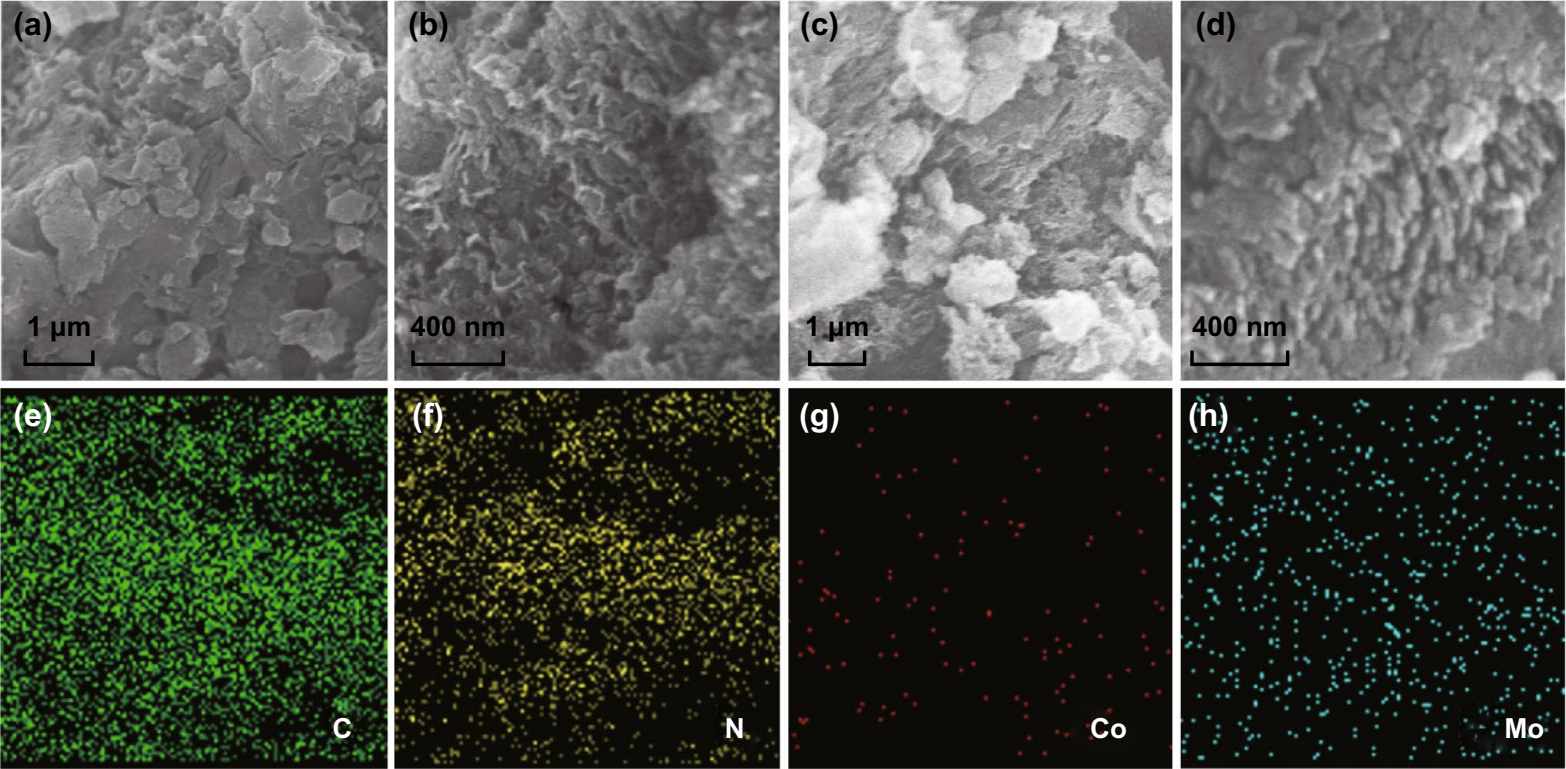
SEM images of **a** P-CN, **b** Co-CN-5, **c** Mo-MCN-2, **d** Co/Mo-MCN-5, **e–h** elemental mapping for Co/Mo-MCN-5

The morphology of P-CN and Co/Mo-MCN-5 were studied by transmission electron microscopy (TEM). P-CN (Fig. 3a, b) exhibits typical sheet-like morphology as other g-C_3_N_4_ materials reported elsewhere. Figure 3c, d reveals the wrinkled and worm-like structure of Co/Mo-MCN-5 with distinct mesoporous channels; the pore size can be estimated as 20–30 nm, which is in accordance with the PSD analysis. Furthermore, no nanoparticles are apparent in the TEM images of Co/Mo-MCN-5; this indicates the absence of metal or metal oxide, which is in accordance with the results of the XRD. The result that mesopores are present in Mo-MCN and absent in Co-CN (in agreement with the N_2_ sorption result; shown in Fig. S6) indicates that the mesopores can be mainly attributed to the utilization of MoS_2_ as a catalyst for decomposing g-C_3_N_4_. SEM measurements were further carried out to analyze the morphology of the as-prepared materials. P-CN exhibits a stacked layer structure, whereas Co-CN-5, Mo-MCN-2, and Co/Mo-MCN-5 consist of a large number of lamellar structures and exhibit wrinkles and irregular folding structures (Fig. 4a–d). This result is in accordance with the high specific surface area of Mo-MCN-2 and Co/Mo-MCN. However, the degree of polymerization and the lamellar structures of Co-CN and Co/Mo-MCN decrease with the increase in the Co content (Figs. S7a-c and S8); this is in agreement with the XRD. The EDS elemental mapping of Co/Mo-MCN-5, Co-CN-5, and Mo-MCN-2 are presented in Figs. 4e–h, S7d–f, and S9; these are applied to verify the presence of doping elements. On the basis of the characterization results, the Co and Mo elements are more likely to be atomic and highly dispersed in the g-C_3_N_4_.

**Fig. 5:**
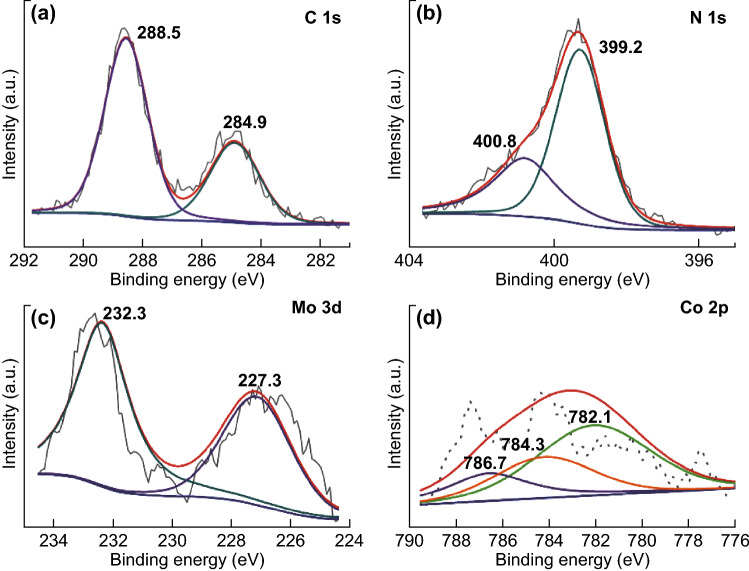
XPS spectra of **a** C1s, **b** N1s, **c** Mo3d, and **d** Co2p for Co/Mo-MCN-5

The elemental valence state and surface composition of the as-prepared materials were further analyzed by XPS. In the case of Co/Mo-MCN-5 (Fig. 5), the C1s exhibits two peaks: at 284.9 and 288.5 eV (Fig. 5a). The former (as a standard carbon) belongs to C–C bonds, and the latter is ascribed to the N=C–N bonds of the g-C_3_N_4_ framework. Figure 5b depicts the high resolution N1s spectrum of Co/Mo-MCN-5. Two peaks at 399.2 and 400.8 eV can be observed; these are ascribed to C=N–C (pyridine nitrogen) and N–C_3_ (tertiary nitrogen), respectively [52, 53]. The Mo3d high resolution spectrum in Fig. 5c can be deconvoluted into two peaks, at 227.3 and 232.3 eV; these can be attributed to the binding energies of Mo3d 5/2 and Mo3d 3/2, respectively, and contributed by the Mo–N bond [54]. As demonstrated in Fig. 5e, the peaks belonging to Co2p can be detected; moreover, the main peaks at 782.1 eV of Co2p 3/2 is attributed to the Co–N bond and is higher than that of Co oxide (779–780 eV) and metallic Co (779 eV) [39, 55]. The peaks at 784.3 and 786.7 eV belong to satellites. Furthermore, the XPS spectra of Co-CN-5 and Co-CN-100 are presented in Fig. S10. These results demonstrate that Co is successfully embedded in the framework of the g-C_3_N_4_ through the formation of Co–N bonds. The formation of the Co–N bonds and Mo–N bonds is likely to be favorable for photocatalytic activity. The Co contents are 0.042% and 0.081% for Co-CN-5 and Co/Mo-MCN-5, respectively, as determined by ICP-AES; the Mo contents are 0.095% and 0.058% for Mo-MCN-2 and Co/Mo-MCN-5, respectively. The contents of Co and Mo for the other materials are presented in Table S2. The XPS spectrum of S2p of Co/Mo-MCN-5 was carried out to verify that S atoms are absent in this case (Fig. S11).

Elemental analysis data of all the as-prepared materials are exhibited in Table S3. The C/N ratio is 0.65 for Mo-MCN and 0.64 for Co/Mo-MCN; these values are marginally lower than that of P-CN (0.68). This implies that Co- and/or Mo-doping inhibits the deamination in thermal polymerization, resulting in further defects [56].

**Fig. 6:**
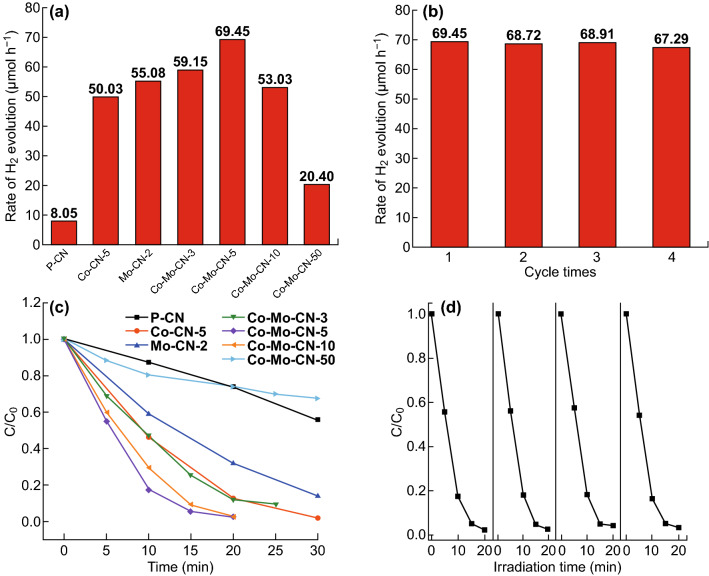
**a** Photocatalytic H_2_ evolution rate of as-prepared materials, **b** cyclic test of photocatalytic H_2_ evolution by Co/Mo-MCN-5, **c** Photocatalytic activities of RhB degradation for as-materials and **d** cyclic test of RhB degradation by Co/Mo-MCN-5

The photocatalytic performance of the obtained materials was evaluated by H_2_ evolution under visible light. All the Co/Mo-MCNs exhibit substantially higher hydrogen evolution rates than that of P-CN (Fig. 6a). The highest hydrogen evolution rate is of Co/Mo-MCN-5: 69.45 μmol h^−1^; the hydrogen evolution rates of Co-CN-5 and Mo-CN-2 are 50.03 and 55.08 μmol h^−1^, respectively. The above three values are higher than that of P-CN’s 8.05 μmol h^−1^ by 8.6, 6.2 and 6.8 times, respectively. Figure S12 demonstrates the H_2_ evolution of Co-CN and Mo-MCN. Notably, the hydrogen evolution rate decreases with the increase in the Co content for Co-CN and Co/Mo-MCN owing to the low crystallinity degree of g-C_3_N_4_. Meanwhile, the long-term stability of hydrogen evolution for Co/Mo-MCN-5 was investigated (Fig. 6b). The result does not reveal apparent decrease in the hydrogen evolution rate after four cycling runs; this indicates that the photocatalyst exhibits high stability. The reused sample was characterized by TEM and IR after the separation from the reaction mixture. The results verify the structure stability of the sample during the photocatalytic process (Fig. S13).

The photocatalytic activity was also assessed by RhB degradation under visible irradiation. P-CN can degrade 19.5% RhB in 15 min; in contrast, Co/Mo-MCN-5 exhibits the highest photocatalytic activity and can degrade 94.7% RhB in 15 min (Fig. 6c). The photocatalytic activities of Co-CN and Mo-MCN are illustrated in Fig. S14a. Co-CN-5 and Mo-MCN-2 could degrade 71.2% and 54.4% RhB in 15 min. The results demonstrate that Co/Mo-MCN exhibits superior photocatalytic performance than those of P-CN, Co-CN, and Mo-MCN. In addition, the photocatalytic activities of Co-CN and Co/Mo-MCN decrease with the increase in the Co content; this can be attributed to the low crystallinity degree of g-C_3_N_4_ according to the XRD results. The cycling test of RhB degradation also verifies the high stability of Co/Mo-MCN-5 (Fig. 6d). Figures S14b and S15 display the kinetic curves of the RhB photodegradation of the as-prepared photocatalysts; these satisfy the pseudo-first-order rate law. The *k* (apparent reaction rate constant) of Co/Mo-MCN-5, Co-CN-5, and Mo-MCN-2 are 0.193, 0.134, and 0.065 min^−1^, which are 10.1, 7.0, and 3.4 times, respectively, than that of P-CN (0.0192 min^−1^).

**Fig. 7:**
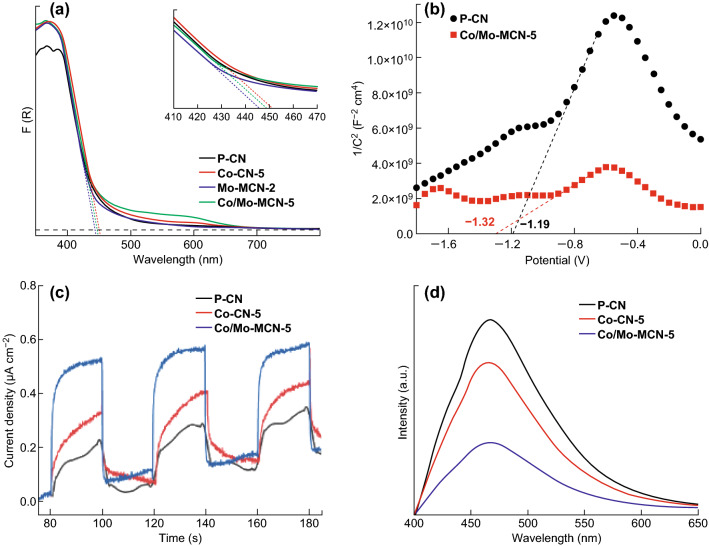
**a** UV–Vis absorption spectra of P-CN, Co-CN-5, Mo-MCN-2, and Co/Mo-MCN-5, **b** Mott–Schottky plots of P-CN and Co/Mo-MCN-5, **c** transient photocurrent responses, and **d** photoluminescence spectra of P-CN, Co-CN-5, and Co/Mo-MCN-5

Furthermore, UV–Vis diffuse reflection spectra were performed to assess the optical absorption of the obtained samples. Co-CN displays enhanced absorption intensity and red-shifted absorption edges from 447 nm of P-CN to 456 nm of Co-CN-100 with the increase in the Co content (Fig. S16), corresponding to the bandgap energy of 2.77 and 2.72 eV, respectively. The absorption regions of Co-CN also extend to approximately 680 nm. The results for the absorption edges and bandgap energy of all the Co-CN materials are listed in Table S4. The decreased bandgap of the Co-CN materials can be attributed to the tuned electronic structure after doping Co into the g-C_3_N_4_ framework. Mo-MCN-1, with a low specific surface area (16.8 m^2^ g^−1^), displays an apparent red shift of the absorption edges to 460 nm (Fig. S17); this is mainly owing to the Mo-doping. However, the decrease in the absorption edge in Mo-MCN-2 and Mo-MCN-4 with the higher specific surface areas (82.4 and 65.1 m^2^ g^−1^) can be observed, which are located at 444 and 442 nm, corresponding to the bandgaps of 2.79 and 2.80 eV, respectively. The increased bandgap can be attributed to the quantum confinement effect caused by the presence of mesopores [57]. As demonstrated in Fig. 7a, the absorption edges of Co/Mo-MCN-5 is 447 nm similar to P-CN; this is a result of the combined effect of the co-doping (of Co and Mo) and the quantum confinement effect. In addition, compared with P-CN, the absorption region of Co/Mo-MCN-5 exhibits an apparent shift up to approximately 680 nm, indicating that more light energy can be absorbed. The extension of the absorption region of Co/Mo-MCN-5 can be ascribed to the charge-transfer transition between the Co ion p-electrons or Mo ion d-electrons and the conduction or valence band of g-C_3_N_4_ [51].

The bandgaps of both P-CN and Co/Mo-MCN-5 are 2.77 eV. Furthermore, the Mott–Schottky measurement was carried out to investigate the conduction band (CB) edge of the materials (Fig. 7b). The positive slopes of the curves indicate the typical n-type characteristics of both the semiconductors. Therefore, the flat band potential can be approximately considered as the CB potential. The upshift of CB can be determined from − 1.19 eV for P-CN to − 1.32 eV for Co/Mo-MCN-5; moreover, Co-CN-5 and Mo-MCN-2 are -1.40 and -1.24 eV, respectively (Fig. S18). Based on the results of the bandgap and CB potential, the valence band (VB) potentials were calculated to be 1.58, 1.36, 1.55, and 1.45 eV for P-CN, Co-CN-5, Mo-MCN-2, and Co/Mo-MCN-5, respectively. This result demonstrates that the increase in CB caused by the bimetal co-doping can dramatically enhance the reduction capability of the photocatalyst; this can significantly improve the photocatalytic performance of hydrogen evolution. Furthermore, the trapping of the photogenerated electrons by transition metals can effectively inhibit the recombination of carriers; this is favorable to direct hole oxidation (RhB degradation) [58].

The photocurrent was carried out to investigate the capability to generate and transfer photogenerated charge carriers under visible light irradiation (Fig. 7c). One can conclude that the photocurrent density of Co/Mo-MCN-5 is significantly higher than that of Co-CN-5 and P-CN. Therefore, Co/Mo-MCN-5 can effectively facilitate the separation of photogenerated charge carriers owing to the mesopore structure, high *S*_BET_, and the formation of Co–N and Mo–N bonds.

The PL spectra are closely related to the recombination of photo-induced electrons and holes. A lower PL intensity generally implies lower recombination of photo-induced charge carries, resulting in an improved photocatalytic performance. Co/Mo-MCN-5 displays significantly lower PL intensity than those of Co-CN-5 and P-CN (Fig. 7d). This can be attributed to the transition of electrons in the mesopores and the trapping of electrons by Co and Mo bonds; these cause the low recombination rate of photogenerated electron–hole pairs, which are favorable to the improvement of photocatalytic activity.

The Co/Mo-MCN-5 conduction band (− 1.32 eV) is more negative than the hydrogen electrode potential (H^+^/H_2_). Therefore, H^+^ can be reduced to H_2_ directly. The radical species trapping experiments were performed to investigate the mechanism of RhB degradation by Co/Mo-MCN-3 by using isopropanol (IPA, as ⋅OH scavenger), p-benzoquinone (BQ, as ⋅O_2_^−^ scavenger), and triethanolamine (TEOA, as h^+^ scavenger). As demonstrated in Fig. S19, the photodegradation efficiency of RhB abruptly decreased from 92.2% to 16.5% when the BQ was added; this indicates that ⋅O_2_^−^ was the predominant active species for RhB degradation by Co/Mo-MCN. In contrast, the negligible inhibition by the addition of IPA indicates that ⋅OH does not positively affect the degradation of RhB. In addition, the degradation efficiency was marginally decreased with the introduction of TEOA. Therefore, these results demonstrate that ⋅O_2_^−^ plays an important role in the degradation of RhB and that h^+^ is also involved in the Co/Mo-MCN catalyst.

The improved photocatalytic activity of Co/Mo-MCN compared with P-CN and mono-metal-doped g-C_3_N_4_ should be ascribed mainly to the synergistic effect of bimetallic doping, including the highly efficient charge separation, the fast mass transfer, and the higher *S*_BET_ and larger pore volumes derived from the abundant mesoporous structure. Moreover, the formation of Co–N and Mo–N bonds by the doping with Co and Mo is another cause. The heteroatom Co or Mo can trap photogenerated electrons to reduce the recombination of charge carriers effectively, thereby increasing the photocatalytic activity. In order to further verify the reasons for the improved photocatalytic performance, the DFT calculations were applied.

**Fig. 8:**
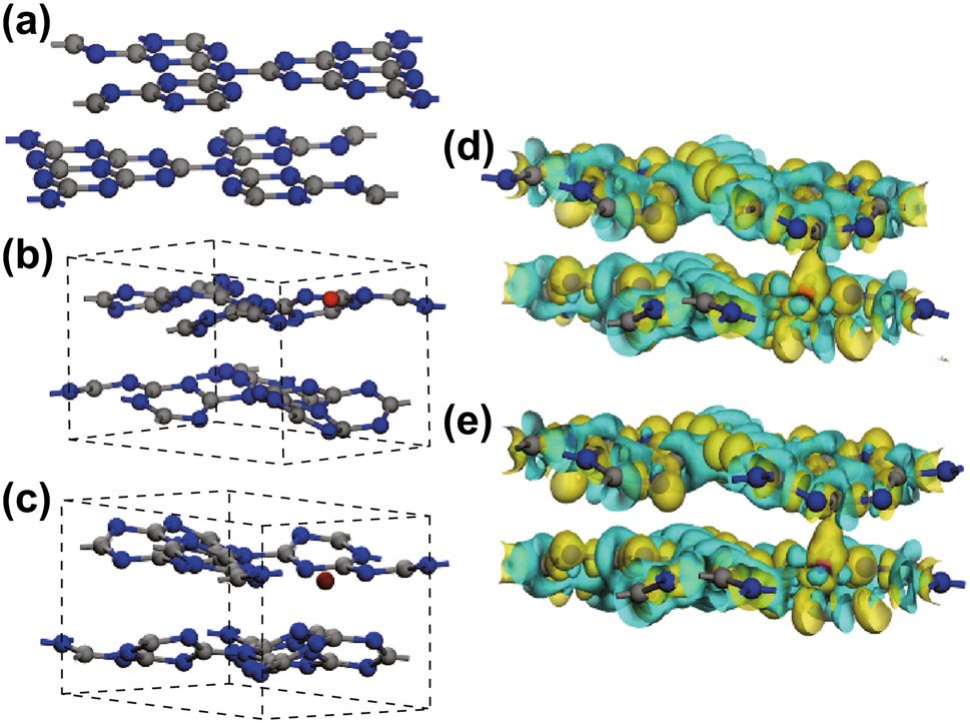
Crystal structure of **a** P-CN, **b** Co-CN, and **c** Mo-CN, and charge density difference of **d** Co-CN and **e** Mo-CN

DFT calculations were utilized to estimate the crystal structures of g-C_3_N_4_ (P-CN), Co-doped g-C_3_N_4_ (Co-CN), and Mo-doped g-C_3_N_4_ (Mo-CN). The crystal structure of P-CN is presented in Fig. 8a. The in-planar repeat period and interlayer distance are estimated to be 7.12 and 3.14 Å, respectively; these results are consistent with previous theoretical results [59, 60]. Based on the P-CN geometry optimization results, the crystal structures of Co-doped and Mo-doped g-C_3_N_4_ were calculated when the doping atom was inserted into the planes and interlayer. Details of the geometry optimization for the different doping sites are presented in Table S5; from this table, one can conclude that the most stable configuration for both Co- and Mo-doped g-C_3_N_4_ is the bridging site. Their final crystal structures are shown in Fig. 8b, c, respectively.

For further analysis of the effects of Co-doping and Mo-doping on the photocatalytic activity, the charge density difference between P-CN and both the metal-doped materials was investigated; and the results are shown in Fig. 8d, e. Either of doped Co and Mo atoms could form chemical bonds between two layers and construct channels to deliver charges, thereby promoting the exciton dissociation [61].

Another factor that substantially affects the photocatalytic performance is the optical absorption characteristics. This can be estimated by the bandgap and optical absorption coefficients [62]. The bandgap determines the optical spectral range where light can be absorbed to excite the photogenerated charge carriers. The optical absorption coefficients determine the optical absorption intensity at different wavelengths.

**Fig. 9:**
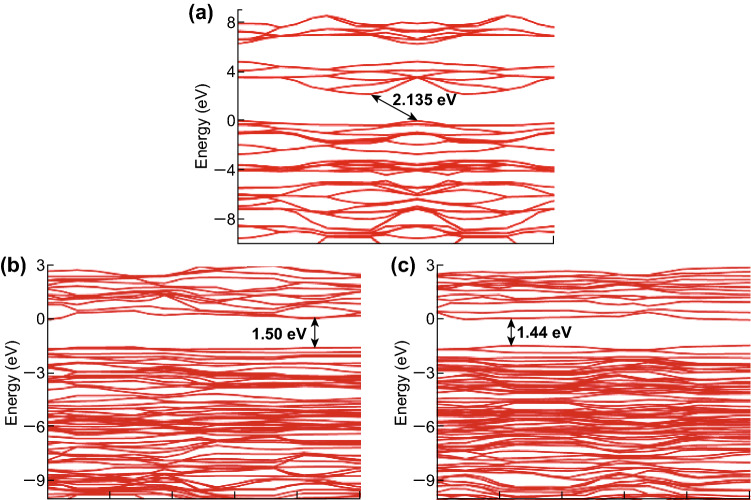
Band structure of **a** P-CN, **b** Mo-CN, and **c** Co-CN

**Fig. 10:**
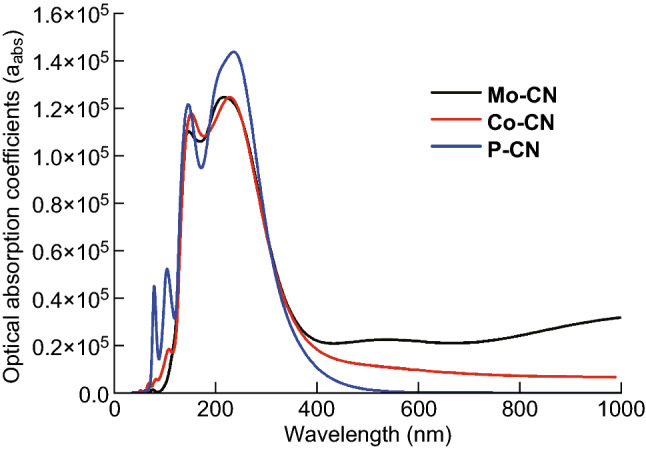
Optical absorption coefficient of P-CN, Co-CN, and Mo-CN

The band structures of P-CN, Co-CN, and Mo-CN are demonstrated in Fig. 9. The bandgap calculated for P-CN is 2.135 eV, which is marginally smaller than the experimental value; this is owing to the constraints posed by DFT. The bandgaps calculated for Mo-CN and Co-CN are 1.50 and 1.44 eV, respectively. Evidently, Co- or Mo-doping could narrow the bandgap and extend the light absorption region that can be used to induce photogenerated electron–hole pairs. From the DFT calculations, the dielectric constants of P-CN, Co-CN, and Mo-CN could also be obtained (Fig. S20). On this basis, the optical absorption coefficient could be calculated using Eq. 1; here, *ε*_1_(ω) and *ε*_2_(ω) are the real and imaginary parts, respectively, of the frequency-dependent dielectric function ε(ω). The calculation results indicate that either of Co- and Mo- doping can enhance the optical absorption intensity in the visible light region from 600 to 1000 nm (Fig. 10), in accordance with the UV–Vis DRS results.

1 $$\alpha = \sqrt 2 \omega \sqrt {\sqrt {\varepsilon_{1}^{2} \left( \omega \right) + \varepsilon_{2}^{2} \left( \omega \right) } - \varepsilon_{1} \left( \omega \right) }$$

The above discussions are mainly on the change in the inherent properties of materials. However, there are certain nanoscale structures in the inner part of the photocatalyst. They can result in near-field effects, which modify the optical absorption properties. Owing to the constraint of optical measurement, the optical characteristics in the inner part of the photocatalyst is challenging to measure. Thus, optical simulation was utilized in this work for a better illustration of the optical absorption characteristics in the inner part of the photocatalyst. Moreover, all the optical simulations in this study were carried out using finite difference time domain (FDTD) method implemented on the FDTD Solutions software developed by Lumerical Solutions.

**Fig. 11:**
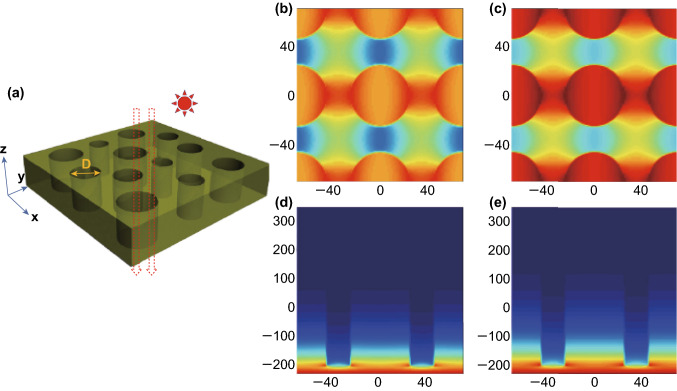
**a** Simulation model of Mo-MCN. D represents mesopores of sizes ranging from 10 to 50 nm, and electric field intensity distribution in x–y plane for **b** P-CN, **c** Mo-MCN, and x–z plane for **d** P-CN, and **e** Mo-MCN (Color changing from blue to red reflects the electric field intensity increasing)

Abundant mesopores with an average size of ca. 25 nm can be observed inside the Mo-MCN photocatalysts in the TEM images; these can influence the optical absorption characteristics. Therefore, the optical characteristics under incident light of wavelengths 300, 500, and 700 nm were calculated for P-CN and Mo-CN. The model adopted in these optical simulations is shown in Fig. 11a. The electric field intensity in the x–y plane could apparently increase at the edge of the pore walls after Mo-doping (Fig. 11b, c). This illustrates that the optical energy gathering at the edge of the pore walls is apparently enhanced after Mo-doping. The electrical field intensity in the x–z plane is also shown in Fig. 11d, e. Moreover, the optical absorption power was calculated according to the electrical field intensity results; the *x*–*z* plane results for P-CN and Mo-CN under incident light of wavelengths 300, 500, and 700 nm are shown in Fig. S21. For the incident wavelength of 300 nm, they exhibit almost identical optical absorption characteristics. However, when the incident wavelength increases to 500 and 700 nm, the optical absorption intensity of Mo-CN is apparently stronger than that of P-CN; this is consistent with the optical absorption coefficient results. Consequently, the optical absorption characteristics can be enhanced by Mo-doping. Based on the above analysis, it can be conjectured that the bimetallic co-doped g-C_3_N_4_ with Co and Mo exhibits stronger light absorption capability than mono-metal doping, resulting in the increased photocatalytic activity.

**Fig. 12:**
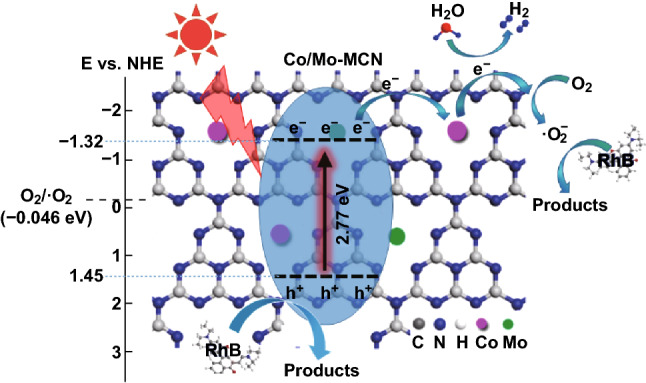
Photocatalytic mechanism scheme of Co/Mo-MCN under visible light irradiation

Based on the above experimental results, a likely mechanism of the degradation of RhB by Co/Mo-MCN is proposed. As demonstrated in Fig. 12, the Co/Mo-MCN can apparently extend the visible light absorption region and absorb more light energy under visible light irradiation; thereby, more active particles could be generated. Then, the photogenerated electrons in the CB of the co-doped material can be trapped by the doped metal (Co and Mo). Subsequently, these electrons react with the dissolved O_2_ in the system to form ⋅O^2−^, which can function as an active species to degrade RhB. However, photogenerated holes cannot react with -OH or H_2_O to produce hydroxyl radicals ⋅OH because the reduction potential of Co/Mo-MCN (+1.53 eV) is more negative than that of OH/OH − (+1.99 eV). Therefore, hydroxyl radicals are mainly derived from the reaction of superoxide radicals with aqueous media. Meanwhile, photogenerated holes remaining in the VB of Co/Mo-MCN can directly oxidize RhB. In addition, the H_2_ production from water-splitting is a process of electron reduction. Considering the above analysis, Co/Mo-MCN’s mesoporous structure and higher specific surface area can promote efficient separation of photogenerated electrons and holes and accelerate mass transfer, which are favorable to the photoreaction process. After the introduction of Co and Mo, the numerous metal–N bonds formed can be considered as a trapping center of electrons that enhances the separation of electron–hole pairs. Therefore, the bimetal-doped Co/Mo-MCN exhibits remarkable photocatalytic activity.

## Conclusions

In summary, Co/Mo-MCN with high specific surface area was synthesized via template-free method. DFT calculations and FDTD simulations revealed that the bimetal co-doping can change the inherent optical properties of the material, thus extending the absorption region and increasing the absorption intensity. Moreover, it was revealed that the intrinsic mesoporous channels and wrinkle structure can improve the light absorption capability at the edge of the pore and reduce reflectance. Co/Mo-MCN exhibited superior photocatalytic performance for RhB degradation and hydrogen evolution than that of P-CN and mono-metal-doped g-C_3_N_4_. The improved photocatalytic performance is mainly ascribed to the synergistic effect of Co and Mo bimetallic doping, which resulted in larger specific surface area, narrowed bandgap, more negative CB potential, and abundant metal–N bonds. These brought about extended visible light absorption region, stronger reduction capability, improved separation efficiency of charge carriers, and accelerated mass transfer. This study provides a fresh perspective for the design and synthesis of high performing photocatalysts with suitable electronic structures and high specific surface areas by applying bimetallic co-doping.

## Electronic supplementary material

Below is the link to the electronic supplementary material.

Supplementary material 1 (PDF 2064 kb)
