# Supplementary material for: In Situ Preparation and Analysis of Bimetal Co-doped Mesoporous Graphitic Carbon Nitride with Enhanced Photocatalytic Activity
Source: Nanomicro Lett. 2019 Jan 23;11:10. doi: 10.1007/s40820-018-0236-y (PMC7770843; doi:10.1007/s40820-018-0236-y)
Supplement: Supplementary file 1 — Supplementary material 1 (PDF 2064 kb) [file 40820_2018_236_MOESM1_ESM.pdf]

Supporting Information for

In-situ Preparation and Analysis of Bimetal Co-doped Mesoporous Graphitic Carbon Nitride with Enhanced Photocatalytic Activity

Wanbao Wu[1, #], Zhaohui Ruan[2, #], Junzhuo Li[1, #], Yudong Li[1], Yanqiu Jiang[1, *], Xianzhu Xu[1], Defeng Li[1], Yuan Yuan[2, *], Kaifeng Lin[1, *]

[1]School of Chemistry and Chemical Engineering, Harbin Institute of Technology, 92 West Dazhi Street, Nan Gang District, Harbin 150001, People's Republic of China

[2]Key Laboratory of Aerospace Thermophysics, Ministry of Industry and Information Technology, Harbin Institute of Technology, 92 West Dazhi Street, Nan Gang District, Harbin 150001, People's Republic of China

[#]W. Wu, Z. Ruan and J. Li contributed equally to this work

*Corresponding authors. Email: jiangyanqiu@hit.edu.cn (Y. Jiang); yuanyuan83@hit.edu.cn (Y. Yuan); linkaifeng@hit.edu.cn (K. Lin)

S1 Experimental Section

S1.1 Materials

Guanidine hydrochloride, cobalt hexahydrate chloride, and sodium molybdate were all purchased from Aladdin, thiourea was purchased from Sinopharm Chemical Reagent Co., Ltd. The materials were used without further purification.

S1.2 Characterizations

X-ray Diffraction (XRD) measurement was conducted to characterize crystal structure of as-prepared samples on Bruker D8 Advance diffractometer by using Cu Ka radiation. Fourier Transform Infrared (FT-IR) spectra was tested in Perkin Elmer 100 spectrometer. The thermal stability of samples was performed by using thermogravimetric (TG) analysis with NETZSCH Instrument STA 449 F3 Jupiter. The chemical state of elements in the as-prepared samples was studied by X-ray photoelectron spectra (XPS) with PHI 5700 ESCA system equipped with an A1 Kα radiation as a source (hυ = 1486.6 eV). A Hitachi S-4800 model microscope was adoped to observe the morphologies of as-prepared samples. Transmission electron microscopy (TEM) images were obtained on a Topcon 002B. UV–vis diffuse

reflection spectra (BaSO4 as the reflectance standard) was analyzed on Perkin Elmer Lambda 750 spectrophotometer from 200 to 800 nm. The BET surface area and pore-size distribution were recorded on Micromeritics ASAP2020. Meanwhile, the photoluminescence (PL) measurement were performed using PerkinElmer LS-55 with 375 nm as excitation wavelength under liquid nitrogen conditions.

S1.3 Photoelectrochemical (PEC) Measurement

CHI electrochemical station (Model 660D) was performed to investigate the electrochemical performances of material using a three-electrode system, and photocurrent was measured under an intermittent transient visible light irradiation, Ag/AgCl electrode (3 M KCl) and Pt plate were used as reference electrode and counter electrode, respectively, the active area of working electrode is confined to 0.19625 cm^2. The working electrode was prepared through dropping the ink that was fabricated by ultrasonically dispersing appropriate amount of materials in a 5 wt% Nafion alcoholic solution. The material was defined at 0.2 mg cm^{-2}. Na_2SO_4 aqueous solution was used as electrolyte. The light source was a 300 W Xe lamp.

S1.4 DFT Calculations

For further illustration of the effects of doping Co and Mo element on the optical properties and chemical properties of g-C_3N_4 with more detail, the first principle calculations with density functional theory are adopted in this work. All of the calculations with DFT are implemented on CASTEP module. Geometry optimizations for g-C_3N_4 (P-CN), Co doped g-C_3N_4 (Co-CN) and Mo doped g-C_3N_4 (Mo-CN) are performed by treating the exchange-correlation interaction with generalized gradient approximation (GGA) in form of Perdew-Wang (PW91) functional, where a hybrid semi-empirical Ortmann–Bechstedt–Schmidt (OBS) approach was used to correct for Van der Waals (VdW) dispersion forces. Optical properties are calculated with Heyd-Scuseria-Ernzerhof (HSE06) hybrid functional. In DFT for geometry optimization, charge density difference and optical properties for g-C_3N_4, Co doped g-C_3N_4 and Mo doped g-C_3N_4, the self-consistent convergence accuracy is set to be 1×10^{-6} eV atom-1, the convergence criterion for the maximal force on atoms is 0.02 eV $Å^{-1}$, the maximum displacement is 5×10^{-4} Å, the cut off energy are set to be 600 eV, and the stress is less than 0.02 GPa. For geometry optimization, the Monkhorst-Pack k-mesh are all set to be 8×8×6. For optical properties, the Monkhorst-Pack k-mesh are all set to be 3×3×1.

Supplementary Figures

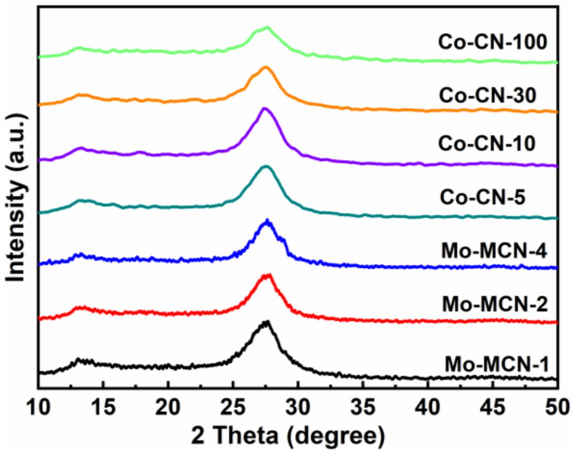

Fig. S1 XRD patterns of Mo-MCN samples and Co-CN

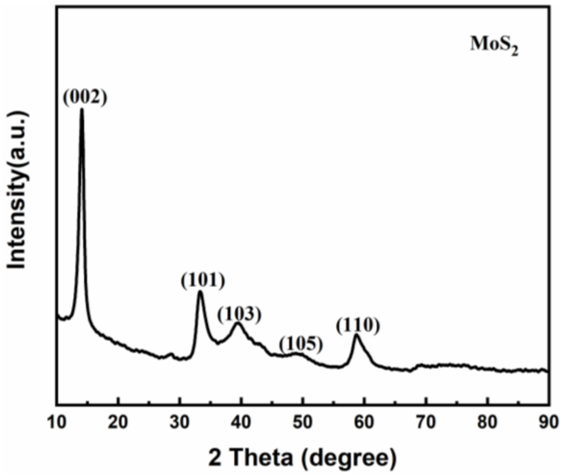

Fig. S2 XRD pattern of MoS_2 nanosheets

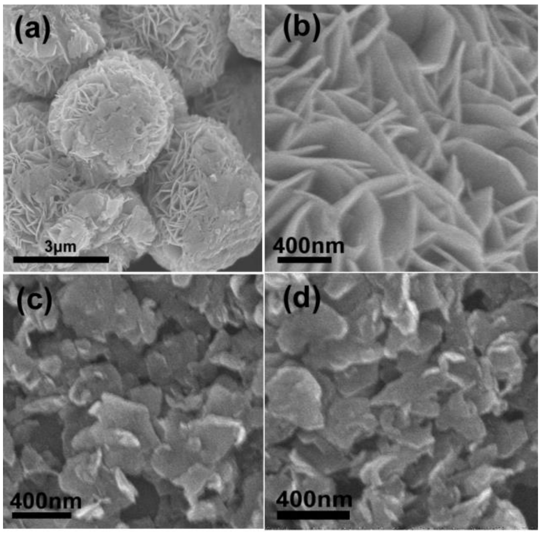

Fig. S3 SEM images of MoS_2 nanoflowers (**a** and **b**) and MoS_2 nanosheets (**c** and **d**)

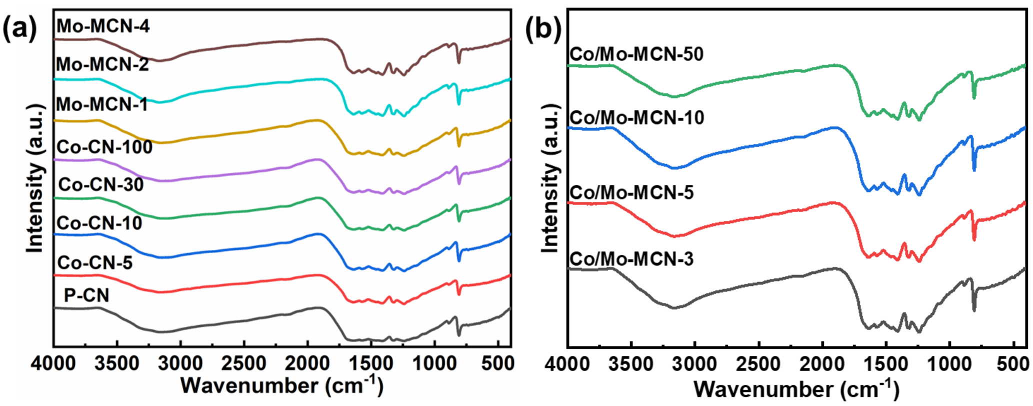

Fig. S4 FI-IR spectra of **a** P-CN, Co-CN, and Mo-MCN and **b** Co/Mo-MCN

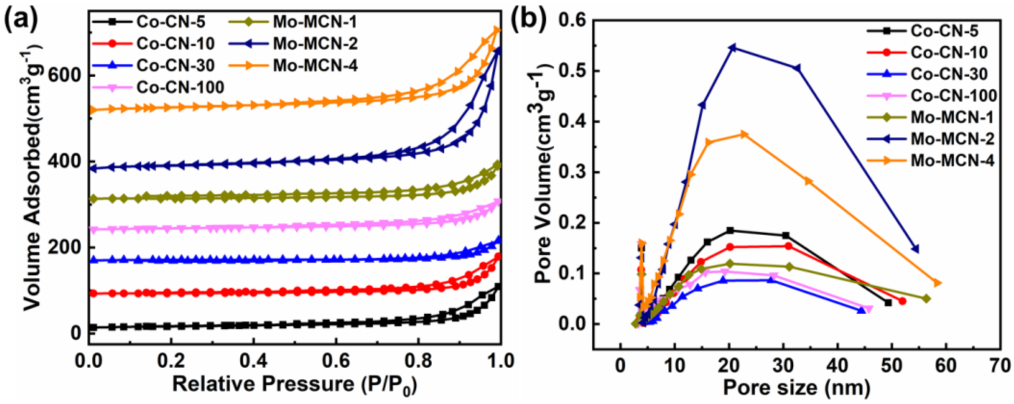

Fig. S5 a Nitrogen adsorption–desorption isotherms and **b** corresponding pore size distribution curves of Co-CN and Mo-MCN

Table S1 physical properties of the as-prepared samples

Sample	S_{BET} (m^2 g^{-1})	Center pore size	Pore Volume (cm^3 g^{-1})
P-CN	16.1	29..2	0.11
Co-CN-5	16.9	20.2	0.11
Co-CN-10	19.9	21.2	0.14
Co-CN-30	18.5	19.3	0.08
Co-CN-100	29.7	16.7	0.12
Mo-MCN-1	16.8	20.2	0.13
Mo-MCN-2	82.4	20.7	0.44
Mo-MCN-4	65.1	22.7	0.31
Co/Mo-MCN-3	61.7	21.9	0.25
Co/Mo-MCN-5	60.6	22.3	0.28
Co/Mo-MCN-10	61.4	21.6	0.32
Co/Mo-MCN-50	65.8	20.7	0.22

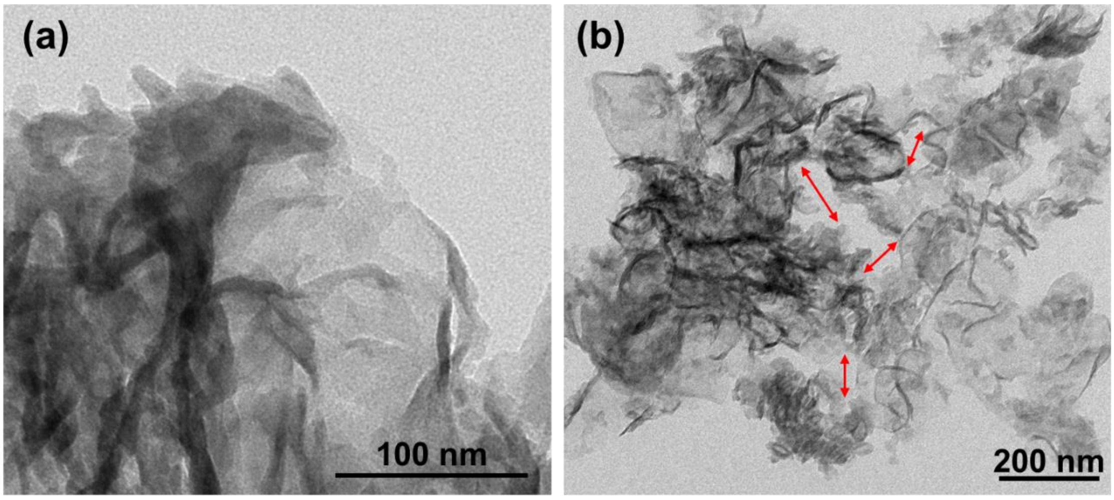

Fig. S6 TEM images of **a** Co-CN-5 and **b** Mo-MCN-2

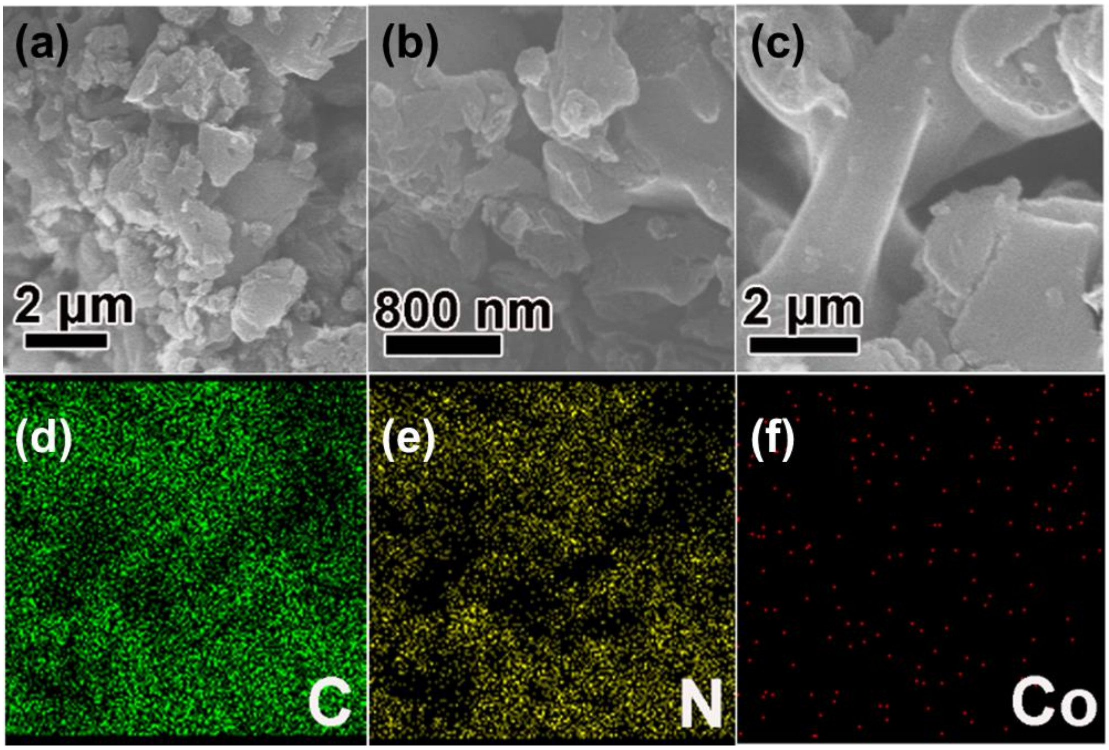

Fig. S7 SEM images of **a** Co-CN-10, **b** Co-CN-30, **c** Co-CN-100 and **d-f** EDS mapping of Co-CN-5

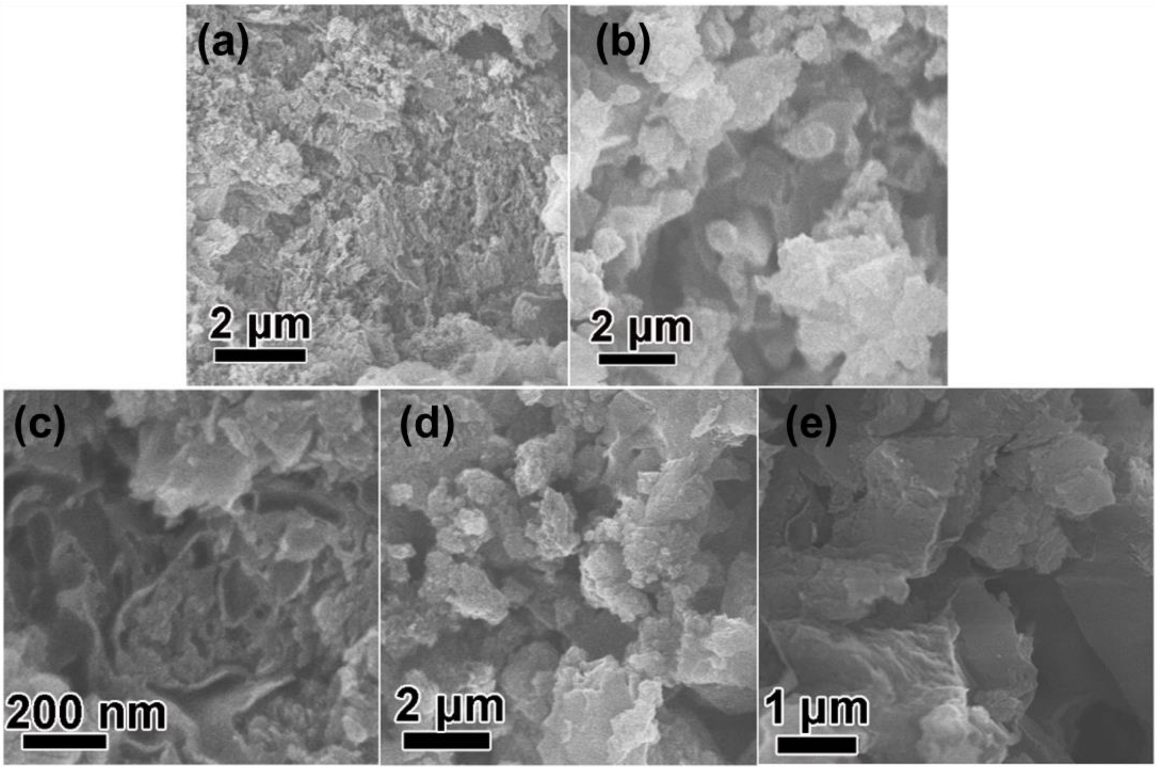

Fig. S8 SEM images of **a** Mo-MCN-1, **b** Mo-MCN-4, **c** Co/Mo-MCN-3, **d** Co/Mo-MCN-10, and **e** Co/Mo-MCN-50

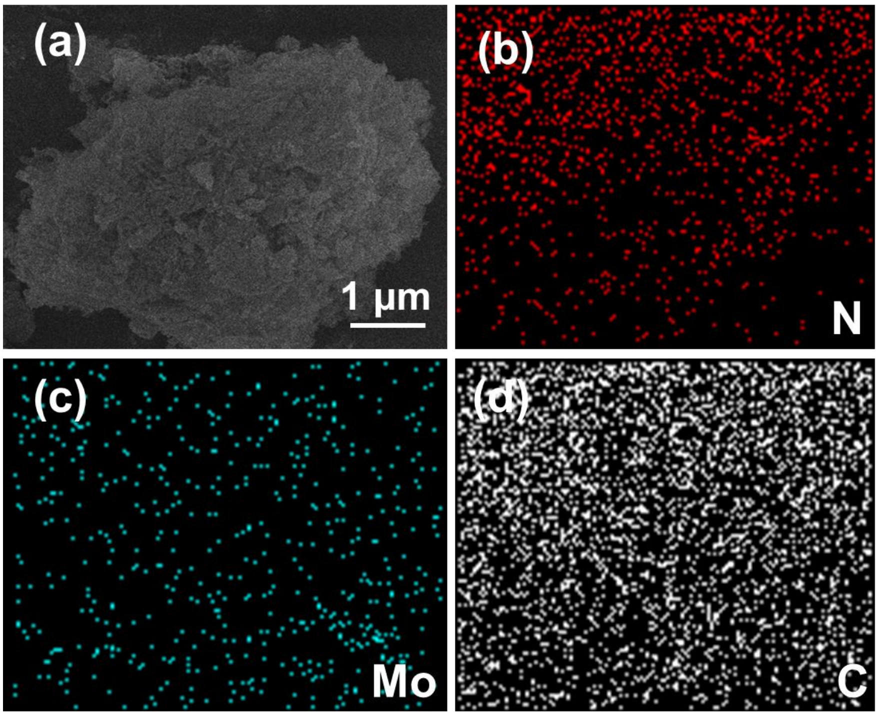

Fig. S9 a SEM image and **b-d** EDS mappings of Mo-MCN-2

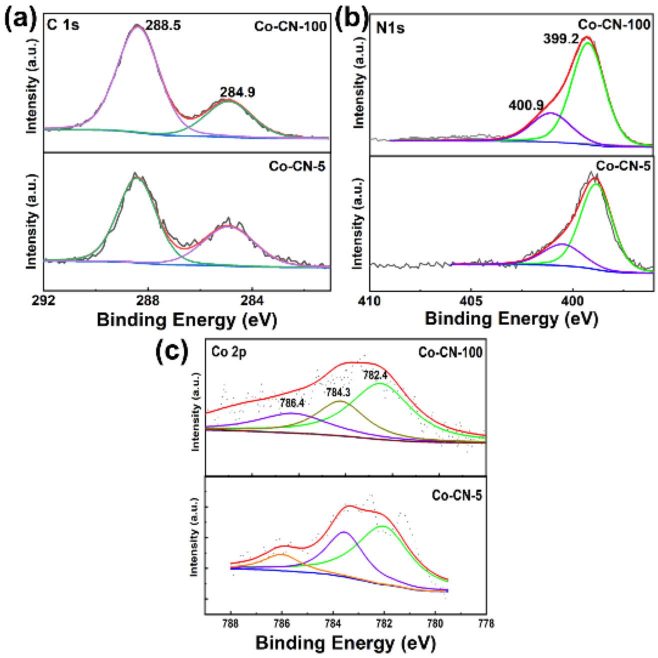

Fig. S10 The XPS spectra of **a** C1s, **b** N1s, and **c** Co2p for Co-CN-5 and Co-CN-100

Table S2 Co content or Mo content of materials

Materials	Co (wt%)	Mo (wt%)
Co-CN-5	0.042	——
Co-CN-10	0.086	——
Co-CN-30	0.210	——
Co-CN-100	0.729	——
Mo-MCN-1	——	0.017
Mo-MCN-2	——	0.095
Mo-MCN-4	——	0.284
Co/Mo-MCN-5	0.081	0.058

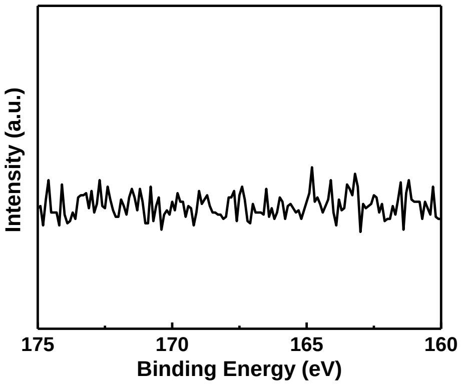

Fig. S11 XPS spectrum of S2p of Co/Mo-MCN-5

Table S3 Elemental composition of P-CN, Co-CN materials, Mo-MCN materials, and Co/Mo-MCN materials obtained from CHN analysis

Materials	Content (wt%)			C/N Ratio (in mole)
	C	N	H	
P-CN	33.46	57.29	2.29	0.68
Co-CN-5	32.79	57.44	2.34	0.66
Co-CN-10	32.67	57.86	2.20	0.66
Co-CN-30	32.58	57.54	2.23	0.66
Co-CN-100	32.01	57.26	2.25	0.65
Mo-MCN-1	32.97	58.07	2.02	0.67
Mo-MCN-2	32.79	58.40	1.99	0.65
Mo-MCN-4	32.05	56.67	2.02	0.65
Co/Mo-MCN-3	32.38	58.64	2.16	0.64
Co/Mo-MCN-5	32.61	59.07	2.09	0.64
Co/Mo-MCN-10	32.32	58.60	2.11	0.64
Co/Mo-MCN-50	30.96	55.32	2.07	0.64

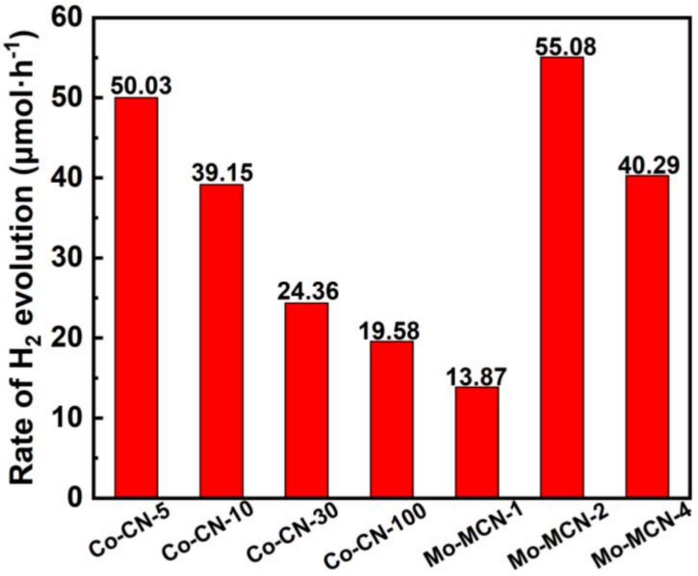

Fig. S12 Photocatalytic H_2 evolution rate of Co-CN materials and Mo-MCN materials

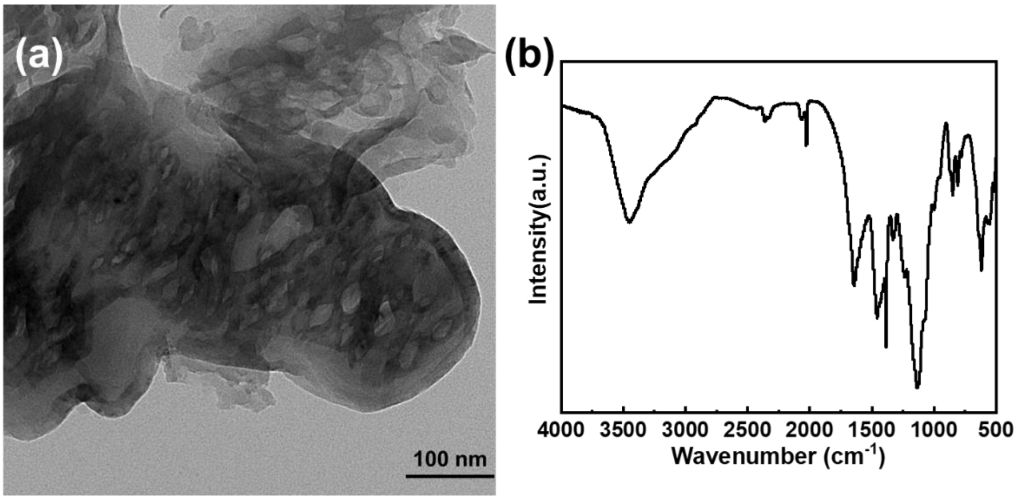

Fig. S13 a TEM image and **b** IR spectrum of reused Co/Mo-MCN-5

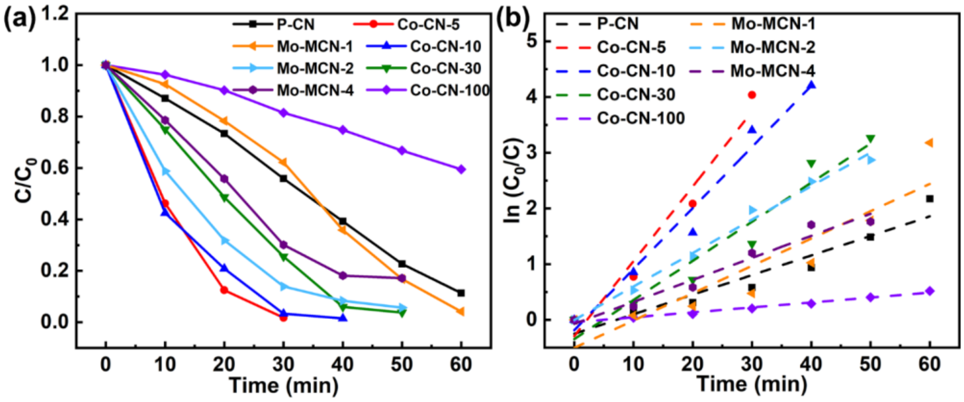

Fig. S14 a Photocatalytic activities and **b** kinetic curves of P-CN, Co-CN and Mo-MCN for degradation of RhB under visible-light irradiation

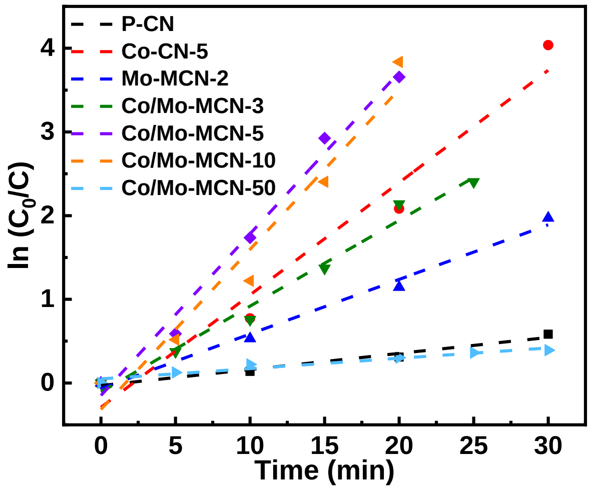

Fig. S15 Kinetic curves of Co/Mo-MCN for degradation of RhB under visible-light irradiation

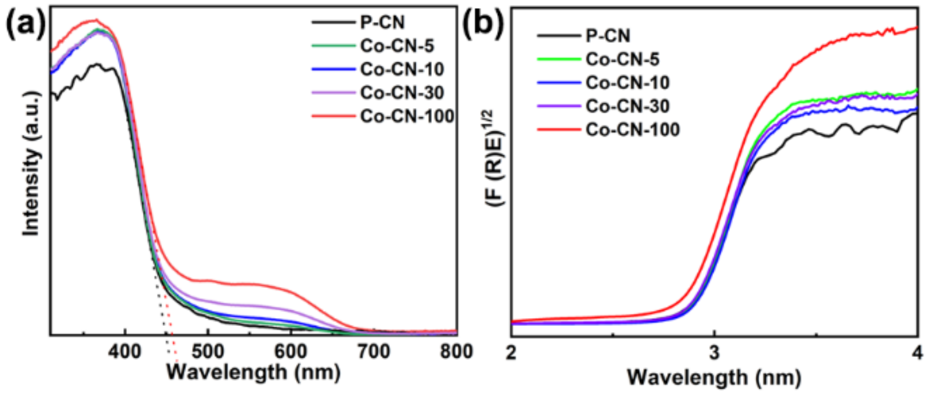

Fig. S16 a UV-Vis diffuse reflectance spectra and **b** band gaps of P-CN and Co-CN

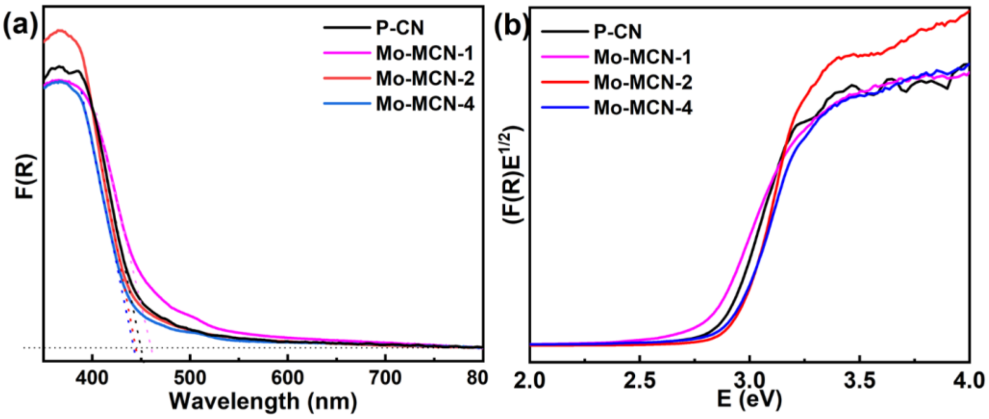

Fig. S17 a UV-Vis diffuse reflectance spectra and **b** band gaps of P-CN and Mo-MCN

Table S4 The optical absorption thresholds and band gaps of materials

Material	Absorption edge (nm)	Bandgap（eV）
P-CN	447	2.77
Co-CN-5	448	2.76
Co-CN-10	450	2.75
Co-CN-30	453	2.74
Co-CN-100	456	2.72
Mo-MCN-1	460	2.69
Mo-MCN-2	444	2.79
Mo-MCN-4	442	2.80
Co/Mo-MCN-5	447	2.77

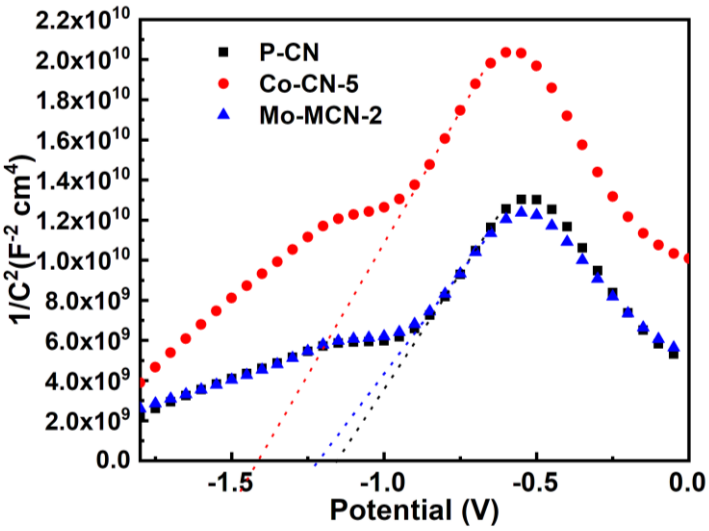

Fig. S18 Mott-Schottky (MS) plots of Co-CN-5 and Mo-MCN-2

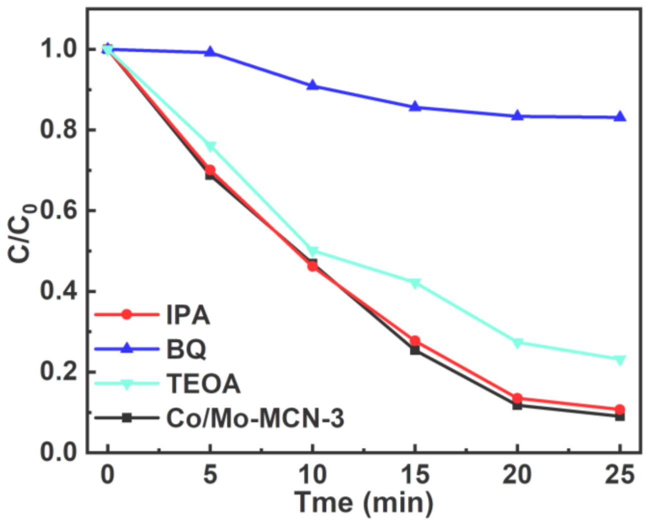

Fig. S19 Species-trapping experiments for degradation RhB over Co/Mo-MCN-3 photocatalysts

Table S5 Details of the geometry optimization for different doping site

Materials	a	b	c	α	β	γ	Final energy (eV)
P-CN	7.13	12.35	6.56	90	90	90	
Co-CN (in bridging)	6.64	6.97	12.10	90	89.93	90	-13443.91
Co-CN (in cave)	7.10	6.41	12.37	90	90	90	-13442.05
Mo-CN (in bridging)	6.65	6.97	12.09	90	90.32	90	-13443.92
Mo-CN (in cave)	6.50	7.10	12.30	90	90	90	-14334.82

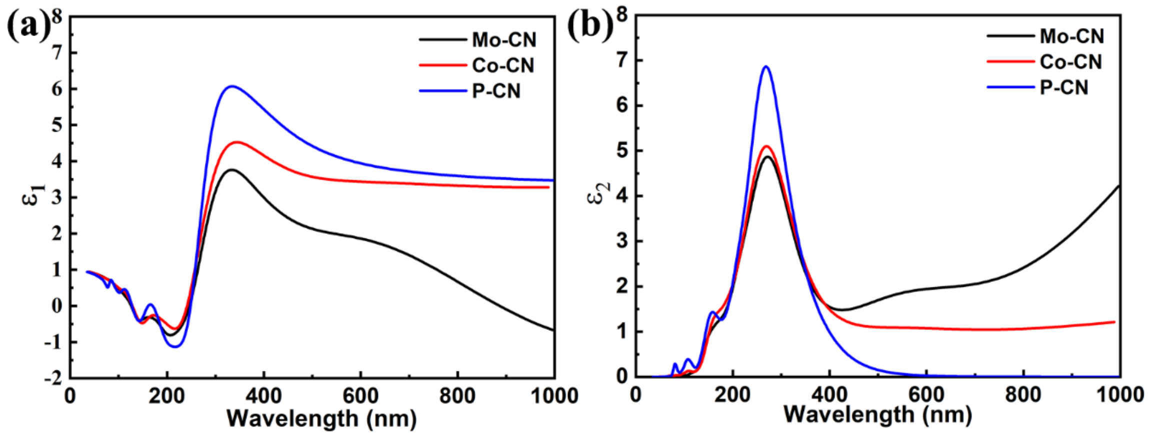

Fig. S20 **a** Real part and **b** imaginary part of the frequency-dependent dielectric function of PCN, Co-CN and Mo-CN

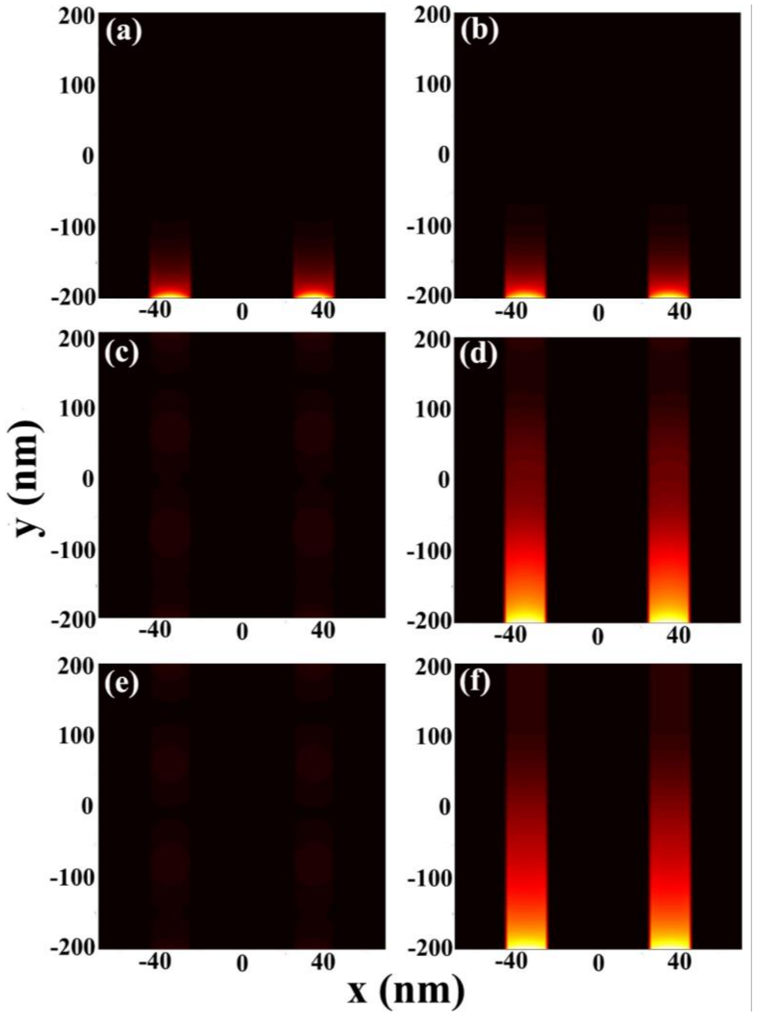

Fig. S21 Optical absorption power of (**a, c** and **e**) P-CN and (**b, d** and **f**) Mo-MCN when the wavelength is 300 nm (**a** and **b**), 500 nm (**c** and **d**) and 700 nm (**e** and **f**)
